# GNN-based trust propagation and intelligent certificate revocation decision mechanism for large-scale IoT networks

**DOI:** 10.1038/s41598-026-43310-4

**Published:** 2026-03-25

**Authors:** Wenlong Han, Muheng Sui, Yi Gao, Pengfei Tao, Donghong Zheng

**Affiliations:** CSG Data Platform and Security (Guangdong) Co., Ltd., Guangzhou, 510220 Guangdong China

**Keywords:** Graph neural network, Trust propagation, Certificate revocation, Internet of things, Graph attention mechanism, Network security, Engineering, Mathematics and computing

## Abstract

The rapid expansion of Internet of Things deployments has introduced significant challenges in trust management and certificate lifecycle administration. Traditional Public Key Infrastructure mechanisms struggle with the scalability and responsiveness demands of large-scale IoT environments. This paper proposes a graph neural network-based framework that integrates trust propagation with intelligent certificate revocation decision-making. We develop a graph attention-based trust propagation model that captures relational dynamics among IoT devices through multi-head attention mechanisms with explicit temporal decay factors. Additionally, we design an adaptive revocation decision algorithm that synthesizes trust embeddings, behavioral anomaly indicators, and topological features to generate risk scores for certificate management. Experimental evaluation across networks comprising up to 102,400 devices demonstrates that our approach achieves trust propagation accuracy exceeding 89% and revocation decision F1 scores of 0.904, with median response latency under five seconds. The proposed framework outperforms the evaluated baseline methods, including traditional reputation-based approaches and standard graph convolutional networks, in both accuracy and computational efficiency within the considered experimental settings, providing a practical solution for securing large-scale IoT infrastructures.

## Introduction

The proliferation of Internet of Things (IoT) devices has fundamentally transformed how digital infrastructures operate across industrial, healthcare, and smart city domains. Current estimates suggest that billions of interconnected devices now exchange sensitive data through heterogeneous communication channels, creating unprecedented security management challenges^1^. Within this expansive ecosystem, trust establishment and certificate lifecycle management have emerged as critical concerns that demand sophisticated solutions beyond conventional approaches.

Traditional Public Key Infrastructure (PKI) systems, originally designed for relatively static network environments, struggle to accommodate the dynamic nature of modern IoT deployments. Certificate revocation mechanisms, in particular, face substantial scalability bottlenecks when applied to networks comprising thousands or even millions of resource-constrained devices^2^. The inherent latency associated with Certificate Revocation Lists (CRLs) and Online Certificate Status Protocol (OCSP) queries introduces unacceptable delays in time-sensitive applications, potentially leaving compromised devices operational during critical windows of vulnerability.

Trust propagation in IoT networks presents its own set of intractable difficulties. Devices frequently join and leave network clusters, establish transient communication links, and operate under varying resource constraints. These characteristics render static trust models inadequate for capturing the fluid relationships that define modern IoT ecosystems^3^. Moreover, the heterogeneity of device capabilities—ranging from computationally powerful edge gateways to severely constrained sensor nodes—complicates the implementation of uniform trust assessment protocols.

Research efforts addressing these challenges have proceeded along several parallel trajectories. Graph-theoretic approaches to network security have gained considerable traction, with investigators recognizing that IoT topologies naturally lend themselves to graph-based representations^4^. The relational structure inherent in device communication patterns encodes valuable information about trust relationships, behavioral anomalies, and potential security threats. Graph Neural Networks (GNNs), capable of learning representations that capture both node attributes and structural properties, offer a promising framework for extracting actionable insights from these complex relational datasets^5^.

Within the security domain specifically, GNN architectures have demonstrated remarkable success in intrusion detection, malware classification, and anomaly identification tasks^6^. The message-passing paradigm underlying most GNN variants aligns naturally with the distributed nature of trust information flow in networked systems. However, direct application of existing GNN models to IoT trust management remains underexplored, with most studies focusing on enterprise networks or social computing scenarios rather than resource-constrained embedded environments.

Parallel developments in IoT-specific trust evaluation have yielded numerous reputation-based and behavior-driven assessment frameworks^7^. These approaches typically aggregate historical interaction data to derive trust scores, yet they often fail to exploit the rich structural information embedded in network topologies. Furthermore, computational complexity concerns frequently limit their applicability to small-scale deployments, as trust calculation overhead grows prohibitively with network size^8^.

Certificate revocation research has similarly evolved to address IoT-specific constraints. Lightweight revocation schemes, blockchain-based approaches, and distributed consensus mechanisms have all been proposed as alternatives to centralized PKI models^9^. Despite these advances, existing solutions generally treat revocation as an isolated security function rather than integrating it with broader trust assessment frameworks. This separation prevents systems from leveraging trust dynamics to inform revocation decisions proactively.

Several critical gaps persist across these research streams. First, computational efficiency remains problematic—existing trust propagation algorithms exhibit polynomial or worse time complexity with respect to network size, rendering them impractical for large-scale deployments^10^. Second, trust assessment accuracy degrades significantly in highly dynamic environments where device behaviors and network conditions fluctuate rapidly. Third, revocation decision-making typically operates reactively, responding to security incidents only after damage has occurred rather than anticipating threats based on evolving trust patterns^11^.

These limitations motivate our investigation into integrated approaches that combine the representational power of graph neural networks with intelligent decision-making mechanisms tailored for IoT environments. We contend that treating trust propagation and certificate revocation as interconnected processes—rather than independent functions—enables more responsive and accurate security management.

This paper presents a GNN-supported trust propagation framework coupled with an intelligent certificate revocation decision mechanism designed specifically for large-scale IoT deployments. Our approach makes several distinct contributions to the field. We develop a novel graph representation scheme that captures both device attributes and interaction patterns within a unified structure amenable to neural network processing. We propose an efficient trust propagation model based on graph attention mechanisms that achieves linear computational scaling while maintaining high assessment accuracy. Additionally, we introduce a predictive revocation decision module that anticipates certificate compromise based on trust trajectory analysis, substantially reducing response latency compared to reactive approaches^12^.

The theoretical significance of this work lies in establishing a principled framework for integrating graph-based learning with trust management theory. From a practical standpoint, our methods enable security administrators to manage certificate lifecycles intelligently across large device populations without sacrificing responsiveness or accuracy. The remainder of this paper elaborates our proposed architecture, presents experimental validation across diverse scenarios, and discusses implications for future IoT security research.

## Related theory and technical foundation

### Graph neural network fundamentals

Graph Neural Networks emerged from the need to process data that inherently resists representation in regular grid structures. Unlike images or sequential text, relational data embedded in graphs demand architectures capable of handling irregular connectivity patterns and variable neighborhood sizes^13^. The evolution of GNN methodologies has progressed remarkably since early spectral approaches first demonstrated the feasibility of extending convolutional operations to graph domains.

Graph Convolutional Networks (GCNs) introduced a simplified yet powerful layer-wise propagation rule. The standard GCN update mechanism can be expressed as follows:

1 $${H}^{(l+1)}=\sigma ({\widetilde{D}}^{-\frac{1}{2}}\widetilde{A}{\widetilde{D}}^{-\frac{1}{2}}{H}^{(l)}{W}^{(l)})$$

where $\widetilde{A}$ denotes the adjacency matrix with self-loops, $\widetilde{D}$ represents the corresponding degree matrix, ${H}^{(l)}$ contains node representations at layer $l$, and $\sigma$ indicates a nonlinear activation function^14^. This formulation enables efficient neighborhood aggregation through sparse matrix operations.

Graph Attention Networks (GATs) refined this approach by introducing adaptive weighting of neighbor contributions. The attention coefficient between nodes $i$ and $j$ follows:

2 $$\frac{{\alpha }_{i}j=}{exp\left(LeakyReLU\left({a}^{T}\left[W{h}_{i}|W{h}_{j}\right]\right)\right){\Sigma }_{k\in {N}_{i}}exp\left(LeakyReLU\left({a}^{T}\left[W{h}_{i}|W{h}_{k}\right]\right)\right)}$$

This attention mechanism allows nodes to differentially weight incoming messages based on learned relevance criteria^15^.

The Message Passing Neural Network (MPNN) framework generalizes these architectures through an abstract formulation:

3 $${h}_{v}^{(t+1)}={U}_{t}({h}_{v}^{(t)},\sum_{u\in \mathcal{N}(v)}{M}_{t}({h}_{v}^{(t)},{h}_{u}^{(t)},{e}_{vu}))$$

where ${M}_{t}$ and ${U}_{t}$ represent message and update functions respectively^16^. This abstraction encompasses most existing GNN variants while providing flexibility for domain-specific customization.

Node embeddings generated through these architectures encode both attribute information and structural context. Graph-level representations, obtained via pooling or hierarchical aggregation, enable classification and regression tasks on entire graphs^17^. The capacity to capture non-Euclidean relationships makes GNNs particularly suited for network security applications, where communication topologies and trust relationships naturally form complex graph structures^18^.

### IoT device trust evaluation models

Trust in IoT environments encompasses multiple interrelated dimensions that collectively characterize device reliability. These attributes typically include communication integrity, service quality consistency, resource availability, and historical behavioral compliance^19^. The multifaceted nature of trust necessitates evaluation frameworks capable of synthesizing diverse evidence sources into coherent assessments.

Direct trust derives from firsthand interaction experiences between devices. A common formulation computes direct trust through weighted aggregation of observed outcomes:

4 $${T}_{direct}(i,j)=\frac{\sum_{k=1}^{n}{w}_{k}\cdot {s}_{k}}{\sum_{k=1}^{n}{w}_{k}}\cdot {\lambda }^{\Delta t}$$

where ${s}_{k}$ represents the satisfaction score of the $k$-th interaction, ${w}_{k}$ denotes its corresponding weight, and ${\lambda }^{\Delta t}$ introduces temporal decay to reflect trust degradation over time^20^. This decay factor proves essential—trust established through past interactions should diminish if not reinforced by recent evidence.

Indirect trust, conversely, relies on third-party recommendations and reputation propagation. The challenge lies in appropriately discounting information that traverses multiple intermediaries. A typical indirect trust computation follows:

5 $${T}_{indirect}\left(i,j\right)=\sum_{m\in {\mathcal{P}}_{ij}^{\left(k\right)}}T\left(i,m\right)\cdot T\left(m,j\right)\cdot {\delta }^{\left|path\right|}, \left|path\right|\le k$$

Here, ${\mathcal{P}}_{ij}^{\left(k\right)}$ represents the set of recommending paths constrained to a maximum of $k$ hops, and ${\delta }^{\left|path\right|}$ penalizes longer propagation chains to account for accumulated uncertainty^21^. In large-scale graphs, enumerating all possible paths between two nodes constitutes an NP-hard problem. To maintain computational tractability, we impose a k-hop neighborhood constraint that restricts path exploration to a bounded depth. Specifically, we employ breadth-first search with early termination at depth $k$ (typically $k=2$ or $k=3$), which yields a time complexity of $O\left(n\cdot {\overline{d}}^{k}\right)$, where $\overline{d}$ denotes the average node degree. For sparse IoT networks where $\overline{d}\ll n$, this approach scales near-linearly with the number of devices. The k-hop limitation aligns naturally with our GNN architecture design, where an $L$-layer network inherently aggregates information from $L$-hop neighborhoods, thereby providing implicit path constraints during trust propagation.

Table 1 summarizes representative trust evaluation approaches documented in the literature, highlighting their methodological foundations and applicability constraints.

**Table 1:** Comparison of typical IoT trust evaluation methods.

Method Category	Trust Source	Computational Complexity	Scalability
Behavior-based	Direct observation	O(n)	High
Reputation aggregation	Network consensus	O(n^2^)	Medium
Recommendation fusion	Third-party endorsement	O(n·m)	Medium
Bayesian inference	Probabilistic evidence	O(n^2^)	Low
Fuzzy logic	Multi-criteria synthesis	O(n·k)	High

As Table 1 shows, behavior-based and fuzzy logic methods exhibit favorable scalability characteristics, whereas Bayesian approaches struggle with computational overhead in dense networks^22^. Reputation aggregation mechanisms, though widely adopted, face vulnerability to collusion attacks where malicious nodes artificially inflate peer ratings^23^.

Trust propagation exhibits three fundamental properties that complicate modeling efforts. Transitivity allows trust to flow through intermediate nodes, yet this transmission inevitably introduces distortion^24^. The decay property ensures that stale trust evidence receives diminished influence. Timeliness demands that evaluation mechanisms respond promptly to behavioral changes—a requirement that proves particularly challenging when network conditions fluctuate rapidly.

Large-scale heterogeneous IoT deployments amplify these challenges considerably. Device diversity complicates the establishment of uniform trust metrics, while massive node populations render centralized aggregation impractical^25^. These constraints motivate our exploration of graph-based approaches capable of distributed trust computation with manageable overhead.

### Digital certificate revocation mechanisms

Public Key Infrastructure provides the cryptographic foundation for device authentication in networked systems, yet certificate validity cannot be assumed throughout the entire certificate lifetime. Compromise events, policy violations, or organizational changes may necessitate premature invalidation—a process that demands efficient and reliable revocation mechanisms^26^.

Certificate Revocation Lists represent the earliest and most straightforward approach to revocation dissemination. Certificate Authorities periodically publish signed lists containing serial numbers of invalidated certificates. The simplicity of this method comes at a cost: CRL sizes grow linearly with accumulated revocations, and the periodic update schedule introduces unavoidable staleness. The revocation window, during which a compromised certificate remains technically valid, can be quantified as:

6 $${W}_{revoke}={t}_{detect}+{t}_{publish}+{t}_{propagate}$$

This window typically spans hours or even days in conventional PKI deployments, creating substantial vulnerability exposure^27^.

Online Certificate Status Protocol addresses the timeliness limitation by enabling real-time validity queries. Relying parties submit certificate identifiers to OCSP responders, receiving immediate status responses. However, this approach introduces its own complications—query overhead scales with verification frequency, and responder availability becomes a critical dependency. Privacy concerns also arise since OCSP queries reveal browsing patterns to third parties^28^.

Certificate Transparency emerged as a complementary mechanism focused on detecting mis-issuance rather than managing revocation directly. By maintaining append-only logs of issued certificates, CT enables retrospective auditing but offers limited utility for real-time revocation scenarios.

IoT environments exacerbate these challenges dramatically. Resource-constrained devices cannot maintain large CRL caches, nor can they afford the communication overhead of frequent OCSP queries^29^. Battery-powered sensors, intermittent connectivity, and massive device populations collectively demand revocation approaches fundamentally different from enterprise PKI assumptions.

These constraints have motivated research into intelligent revocation decision-making. Rather than treating revocation as a binary administrative action, emerging approaches frame it as a predictive problem—anticipating compromise likelihood based on behavioral indicators and trust dynamics^30^. This perspective aligns naturally with the graph-based trust propagation framework developed in our research.

## GNN-based trust propagation and certificate revocation decision model

### Overall system architecture design

Building upon the theoretical foundations established in the preceding sections, we now present a comprehensive architecture for intelligent trust management and certificate revocation in large-scale IoT deployments. The proposed system integrates graph-based representation learning with real-time decision mechanisms to address the scalability and responsiveness challenges identified earlier.

**Fig. 1:**
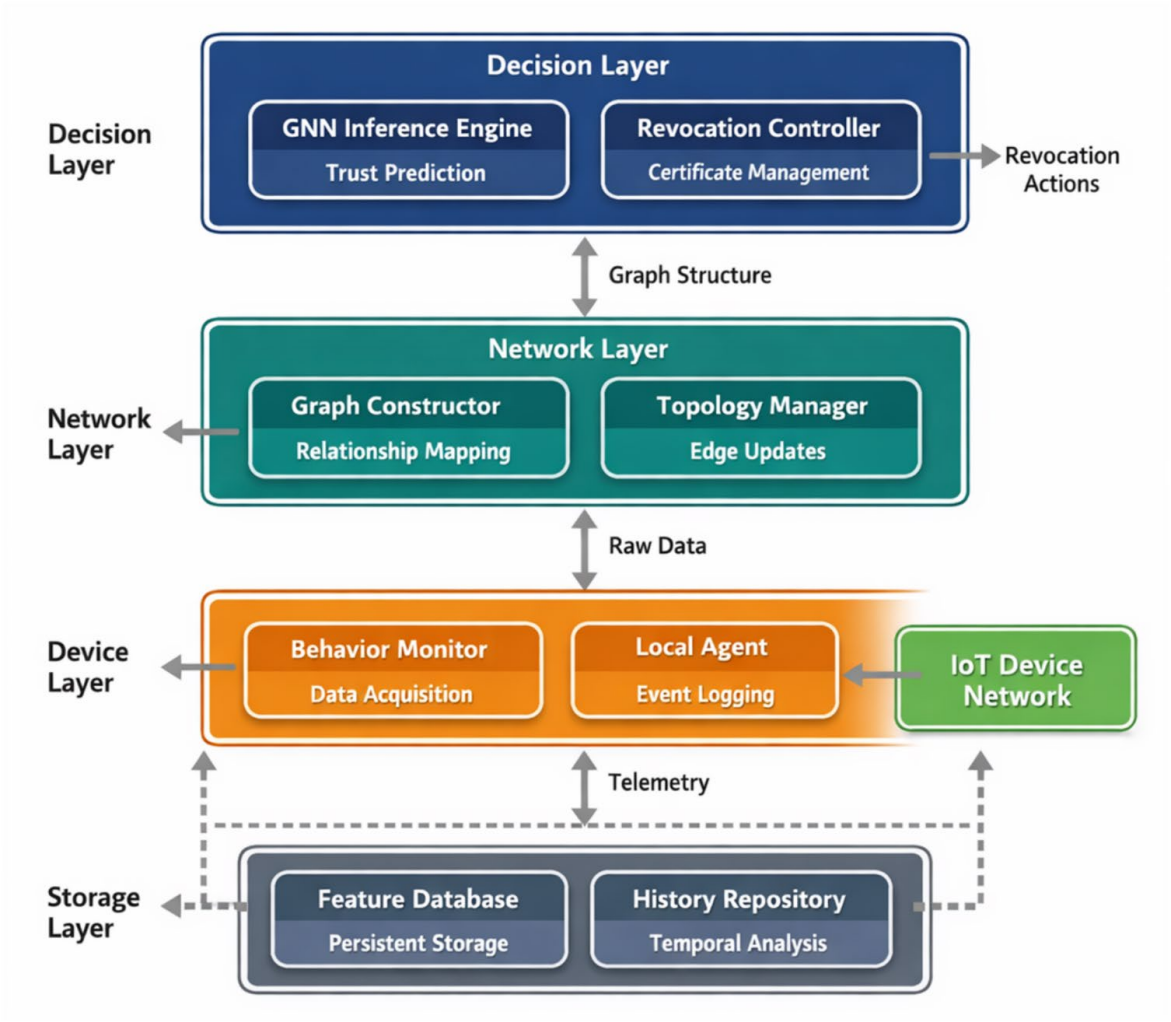
Overall system architecture diagram.

Figure 1 illustrates the overall architecture, which adopts a hierarchical design philosophy to accommodate the inherent heterogeneity of IoT ecosystems.

As shown in Fig. 1, the architecture comprises three interconnected layers, each fulfilling distinct functional responsibilities while maintaining bidirectional information exchange with adjacent layers. The device layer encompasses physical IoT nodes and their local monitoring agents, responsible for behavioral data collection and preliminary feature extraction. The network layer handles trust relationship graph construction and maintains dynamic topology information. The decision layer hosts the GNN-based inference engine and orchestrates revocation actions based on model outputs^31^.

Table 2 provides a detailed breakdown of the functional modules residing within each architectural layer, clarifying their respective roles in the overall workflow.

**Table 2:** Functional module description for each system layer.

Layer	Core Modules	Primary Functions
Device Layer	Behavior Monitor, Local Agent	Data acquisition, event logging
Network Layer	Graph Constructor, Topology Manager	Relationship mapping, edge updates
Decision Layer	GNN Engine, Revocation Controller	Trust inference, certificate management
Storage Layer	Feature Database, History Repository	Persistent storage, temporal analysis

As Table 2 shows, the modular decomposition facilitates independent scaling of computational resources according to deployment requirements. The storage layer, though not explicitly depicted in the hierarchical diagram, provides persistent data services across all functional components^32^.

The graph structure representation constitutes a critical design element. We model the IoT network as a directed attributed graph $\mathcal{G}=(\mathcal{V},\mathcal{E},X,W)$, where $\mathcal{V}$ represents the device node set and $\mathcal{E}$ captures communication relationships. Node attribute vectors encode device-specific characteristics:

7 $${x}_{i}=[{c}_{i},{r}_{i},{\tau }_{i},{b}_{i}^{T}{]}^{T}\in {\mathbb{R}}^{d}$$

Here, ${c}_{i}$ denotes certificate validity status, ${r}_{i}$ represents reputation score, ${\tau }_{i}$ indicates device type encoding, and ${b}_{i}$ contains behavioral feature statistics^33^. Edge weights reflect interaction intensity and historical trust assessments:

8 $${w}_{ij}=\alpha \cdot {f}_{ij}+\beta \cdot {T}_{history}(i,j)$$

where ${f}_{ij}$ quantifies communication frequency and ${T}_{history}(i,j)$ aggregates prior trust evaluations between nodes $i$ and $j$.

The operational workflow proceeds through four sequential stages. Data acquisition modules continuously harvest behavioral observations from distributed device agents. Feature extraction transforms raw telemetry into standardized attribute vectors suitable for graph embedding. The GNN inference engine processes the constructed graph to generate trust predictions and anomaly indicators. Finally, the revocation controller translates model outputs into actionable certificate management decisions, triggering revocation procedures when threat confidence exceeds predefined thresholds^34^. This pipeline operates continuously, enabling near real-time adaptation to evolving network conditions.

### Graph attention-based trust propagation model

The trust propagation mechanism forms the computational core of our proposed system. We develop a specialized graph attention architecture that captures the nuanced dynamics of trust flow across IoT device networks while respecting the physical constraints of distributed deployments.

Formally, we represent the IoT trust network as a directed graph ${\mathcal{G}}^{t}=({\mathcal{V}}^{t},{\mathcal{E}}^{t})$ at time instance $t$, where each node ${v}_{i}\in {\mathcal{V}}^{t}$ corresponds to an authenticated device and edges encode observed communication relationships. The initial trust embedding for node $i$ combines intrinsic device attributes with historical interaction statistics:

9 $${h}_{i}^{(0)}={W}_{init}\cdot {x}_{i}+{b}_{init}$$

where ${W}_{init}\in {\mathbb{R}}^{{d}{\prime}\times d}$ projects the raw feature vector into the latent embedding space^35^.

**Fig. 2:**
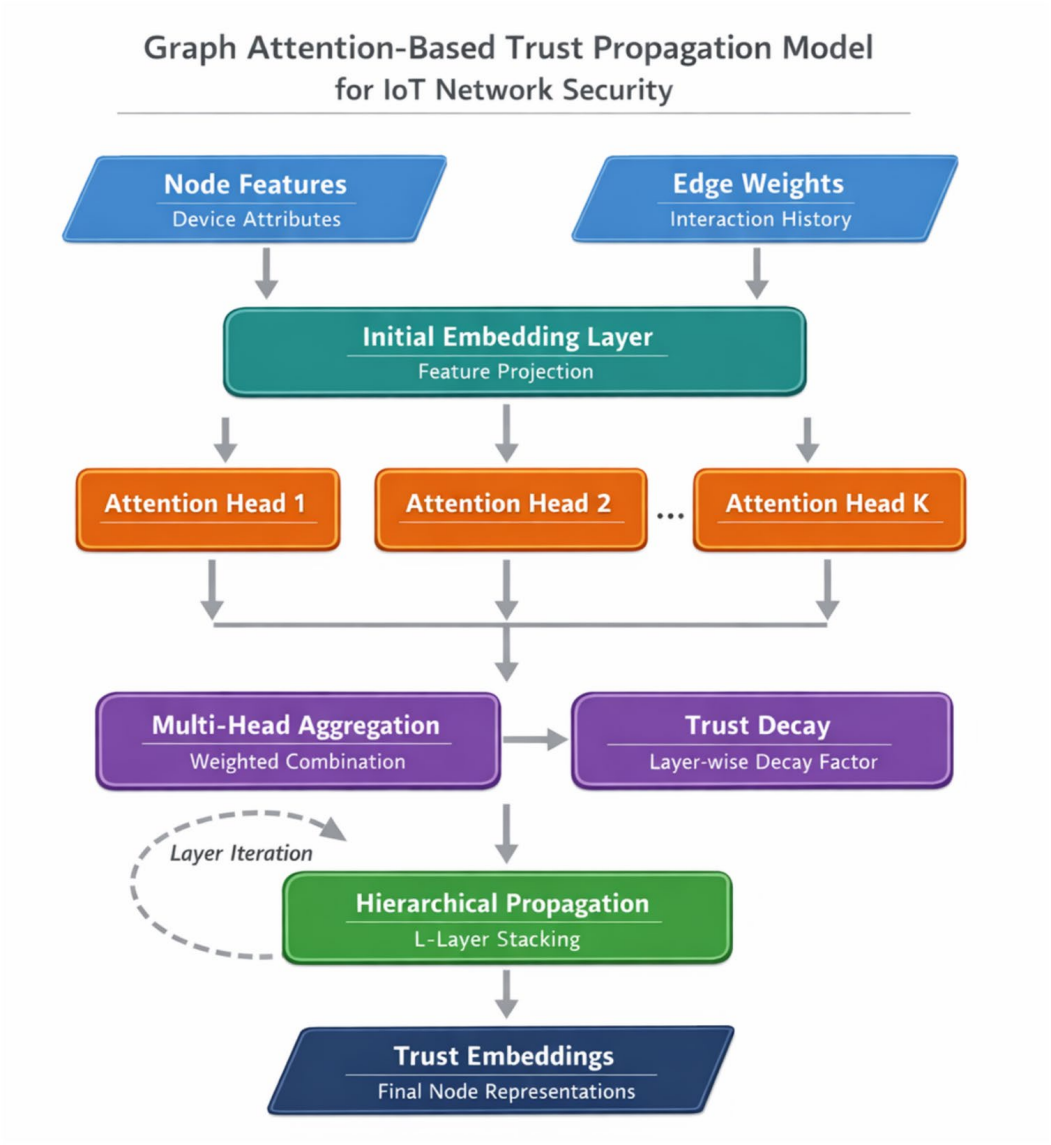
Trust propagation model flowchart.

Figure 2 depicts the complete trust propagation workflow, illustrating how information flows through successive processing stages.

As Fig. 2 demonstrates, the model processes input features through multiple attention layers before generating final trust assessments. The multi-head attention mechanism enables differentiated weighting of neighbor contributions based on learned relevance patterns. For each attention head $k$, we compute pairwise attention coefficients as:

10 $${e}_{ij}^{(k)}=\mathrm{LeakyReLU}\left({a}^{(k)T}[{W}^{(k)}{h}_{i}|{W}^{(k)}{h}_{j}|\phi ({w}_{ij})]\right)$$

The function $\phi (\cdot )$ encodes edge weight information, allowing the model to incorporate existing trust evidence into the attention computation^36^. Normalization across the neighborhood yields the final attention weights:

11 $${\alpha }_{ij}^{(k)}=\frac{\mathrm{exp}({e}_{ij}^{(k)})}{\sum_{m\in {\mathcal{N}}_{i}}\mathrm{exp}({e}_{im}^{(k)})}$$

A critical innovation in our approach involves the explicit modeling of trust decay. Unlike standard attention mechanisms, trust propagation must account for diminishing reliability as information traverses longer paths. We introduce a layer-wise decay factor:

12 $${h}_{i}^{(l+1)}=\sigma \left({\gamma }^{l}\cdot \frac{1}{K}\sum_{k=1}^{K}\sum_{j\in {\mathcal{N}}_{i}}{\alpha }_{ij}^{(k)}{W}^{(k)}{h}_{j}^{(l)}\right)$$

Here, $\gamma \in (\mathrm{0,1})$ controls the decay rate, and $K$ denotes the number of attention heads^37^. This formulation ensures that distant trust evidence receives appropriately discounted influence.

The hierarchical architecture stacks $L$ such layers to enable multi-hop trust propagation. Each layer expands the effective receptive field by one hop, meaning an $L$-layer network can aggregate trust information from devices up to $L$ steps away. The final node representation captures both local behavioral patterns and broader network-level trust context:

13 $${z}_{i}=\mathrm{MLP}\left([{h}_{i}^{(0)}|{h}_{i}^{(1)}|\cdots |{h}_{i}^{(L)}]\right)$$

Table 3 lists the hyperparameter configurations adopted in our implementation, reflecting empirical tuning across diverse network scenarios.

**Table 3:** Model hyperparameter settings.

Parameter	Symbol	Value
Embedding dimension	${d}{\prime}$	128
Number of attention heads	$K$	8
Network depth	$L$	3
Decay factor	$\gamma$	0.85
Learning rate	$\eta$	0.001
Dropout rate	${p}_{drop}$	0.3
Batch size	$B$	256
Weight decay	${\lambda }_{reg}$	5e-4

As Table 3 indicates, we employ three propagation layers with eight attention heads—a configuration that balances expressiveness against computational overhead^38^.

Dynamic graph updates present particular challenges in IoT environments where devices frequently join, depart, or modify their communication patterns. We address this through an incremental update strategy that avoids complete graph reconstruction. When node ${v}_{new}$ enters the network, its initial embedding derives from attribute features alone. Subsequent interactions progressively refine this estimate:

14 $${h}_{new}^{(l)}\leftarrow (1-\mu )\cdot {h}_{new}^{(l)}+\mu \cdot \mathrm{Aggregate}(\{{h}_{j}^{(l-1)}:j\in {\mathcal{N}}_{new}\})$$

The momentum coefficient $\mu$ controls adaptation speed, preventing abrupt embedding shifts while enabling responsive trust updates^39^. Edge deletions trigger localized recomputation limited to affected neighborhoods, maintaining system responsiveness even under high churn conditions.

### Intelligent certificate revocation decision algorithm

Translating trust assessments into actionable revocation decisions requires a principled framework that balances security responsiveness against operational stability. The decision algorithm must process heterogeneous signals—trust embeddings, behavioral anomalies, topological factors—and synthesize them into coherent revocation recommendations.

The input feature vector for device $i$ at decision time $t$ concatenates the GNN-derived embedding with supplementary risk indicators:

15 $${f}_{i}^{t}=[{z}_{i}|\Delta {T}_{i}|{a}_{i}^{t}|{d}_{i}^{out}|{d}_{i}^{in}]$$

Here, ${z}_{i}$ represents the final trust embedding from the propagation model, $\Delta {T}_{i}$ captures recent trust trajectory (the rate of trust change), ${a}_{i}^{t}$ quantifies anomalous behavior intensity, and the degree terms ${d}_{i}^{out}$, ${d}_{i}^{in}$ encode topological centrality. The inclusion of connectivity measures proves essential—highly connected devices warrant more conservative revocation treatment given their potential to disrupt network operations if incorrectly flagged^40^.

The decision output space encompasses three discrete actions: maintain current certificate status, issue a warning with enhanced monitoring, or initiate immediate revocation. We formulate revocation risk as a continuous score that subsequently maps to these categorical outcomes. The risk scoring function combines learned transformations with interpretable factors:

16 $${R}_{i}=\sigma \left({w}_{r}^{T}\cdot \mathrm{MLP}({f}_{i}^{t})+{\beta }_{1}(1-{T}_{i})+{\beta }_{2}\cdot {a}_{i}^{t}+{\beta }_{3}\cdot \frac{{d}_{i}^{out}}{\overline{d}}\right)$$

The sigmoid activation $\sigma (\cdot )$ constrains risk scores to the unit interval, facilitating probabilistic interpretation. The explicit trust complement term $(1-{T}_{i})$ ensures that low-trust devices receive elevated risk assessments regardless of learned feature interactions. The degree normalization term ${d}_{i}^{out}/\overline{d}$ amplifies risk for devices exceeding average connectivity, reflecting their heightened potential for damage propagation^41^.

One persistent challenge in revocation systems involves threshold calibration. Fixed thresholds inevitably perform suboptimally across varying network conditions—during attack campaigns, aggressive thresholds reduce exposure windows, while quiescent periods favor conservative settings to minimize false positives. We address this through an adaptive mechanism that adjusts the revocation threshold based on observed network state:

17 $${\theta }^{t+1}={\theta }^{t}+{\eta }_{\theta }\cdot \left(\frac{F{P}^{t}}{F{P}^{t}+T{N}^{t}}-{\rho }_{target}\right)$$

This update rule drives the false positive rate toward a configurable target ${\rho }_{target}$, automatically tightening or relaxing revocation criteria as conditions evolve. The adaptation rate ${\eta }_{\theta }$ controls responsiveness to observed error patterns.

The final decision logic applies the adaptive threshold with hysteresis to prevent oscillatory behavior:

18 $${D}_{i}=\left\{\begin{array}{cccc}\mathrm{Revoke}& \mathrm{if}{R}_{i}>\theta +\epsilon \, \mathrm{Warning}& \mathrm{if}\theta -\epsilon \le {R}_{i}\le \theta +\epsilon \, \mathrm{Maintain}& \mathrm{if}{R}_{i}<\theta -\epsilon \end{array}\right.$$

The margin parameter $\epsilon$ creates a buffer zone where enhanced monitoring substitutes for immediate action, providing opportunity for additional evidence accumulation before irreversible decisions.

Large-scale deployments necessitate batch processing optimizations to manage computational and administrative overhead. Rather than evaluating devices individually, we aggregate candidates into coherent batches based on temporal proximity and topological clustering. The batch optimization objective minimizes total risk exposure subject to processing constraints:

19 $$\underset{\mathcal{B}}{\mathrm{min}}\sum_{i\in \mathcal{B}}{R}_{i}\cdot {\tau }_{i} s.t. \left|\mathcal{B}\right|\le {B}_{\mathrm{max}}$$

The term ${\tau }_{i}$ represents estimated time-to-compromise, prioritizing devices facing imminent threats within capacity-limited batches^42^. This optimization problem resembles a variant of the 0–1 knapsack problem, for which we employ a greedy approximation algorithm. The detailed procedure is presented in Algorithm 1.

**Algorithm 1:**
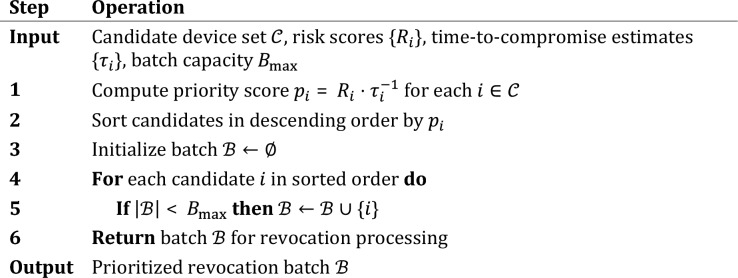
Batch Revocation Optimization.

The time complexity of this algorithm comprises $O\left(n\right)$ for priority computation, $O\left(n\mathrm{log}n\right)$ for sorting, and $O\left(n\right)$ for batch selection, yielding an overall complexity of $O\left(n\mathrm{log}n\right)$ where $n$ denotes the number of candidate devices. When candidate populations exceed 10,000 devices, we adopt a hierarchical processing strategy that partitions candidates by network region before applying the greedy selection within each partition. This approach ensures that revocation resources target the most critical cases when simultaneous processing proves infeasible, while maintaining decision quality comparable to exhaustive individual evaluation.

## Experiments and result analysis

### Experimental Environment and dataset construction

The empirical validation of our proposed framework demands carefully controlled experimental conditions alongside realistic data reflecting genuine IoT operational patterns. We conducted all experiments on a server equipped with dual Intel Xeon Gold 6248R processors, 256 GB RAM, and four NVIDIA A100 GPUs. The software stack comprised Python 3.9, PyTorch 1.12, and the PyTorch Geometric library for graph neural network implementation.

Dataset construction proceeded along two complementary tracks. The primary dataset derives from a smart campus deployment encompassing heterogeneous devices—environmental sensors, surveillance cameras, access control terminals, and edge computing nodes. We collected device interaction logs spanning six months, capturing communication patterns, authentication events, and behavioral telemetry. To ensure sufficient representation of adversarial scenarios, we augmented this foundation with synthetically generated attack traces following established threat models^43^.

Ground truth labels were generated through a systematic procedure reflecting both observed behaviors and injected attack traces. Trust labels adopt continuous values in the range $\left[\mathrm{0,1}\right]$, computed from device historical interaction success rates, protocol compliance metrics, and response time consistency measurements. Specifically, trust ground truth combines three components: communication reliability (ratio of successful message exchanges), service quality consistency (variance in response latencies), and authentication compliance (frequency of valid credential presentations). Compromise labels are binary $\{\mathrm{0,1}\}$, where a value of 1 indicates that a device has been marked as compromised based on either confirmed attack injection or detected malicious behavior patterns.

**Fig. 3:**
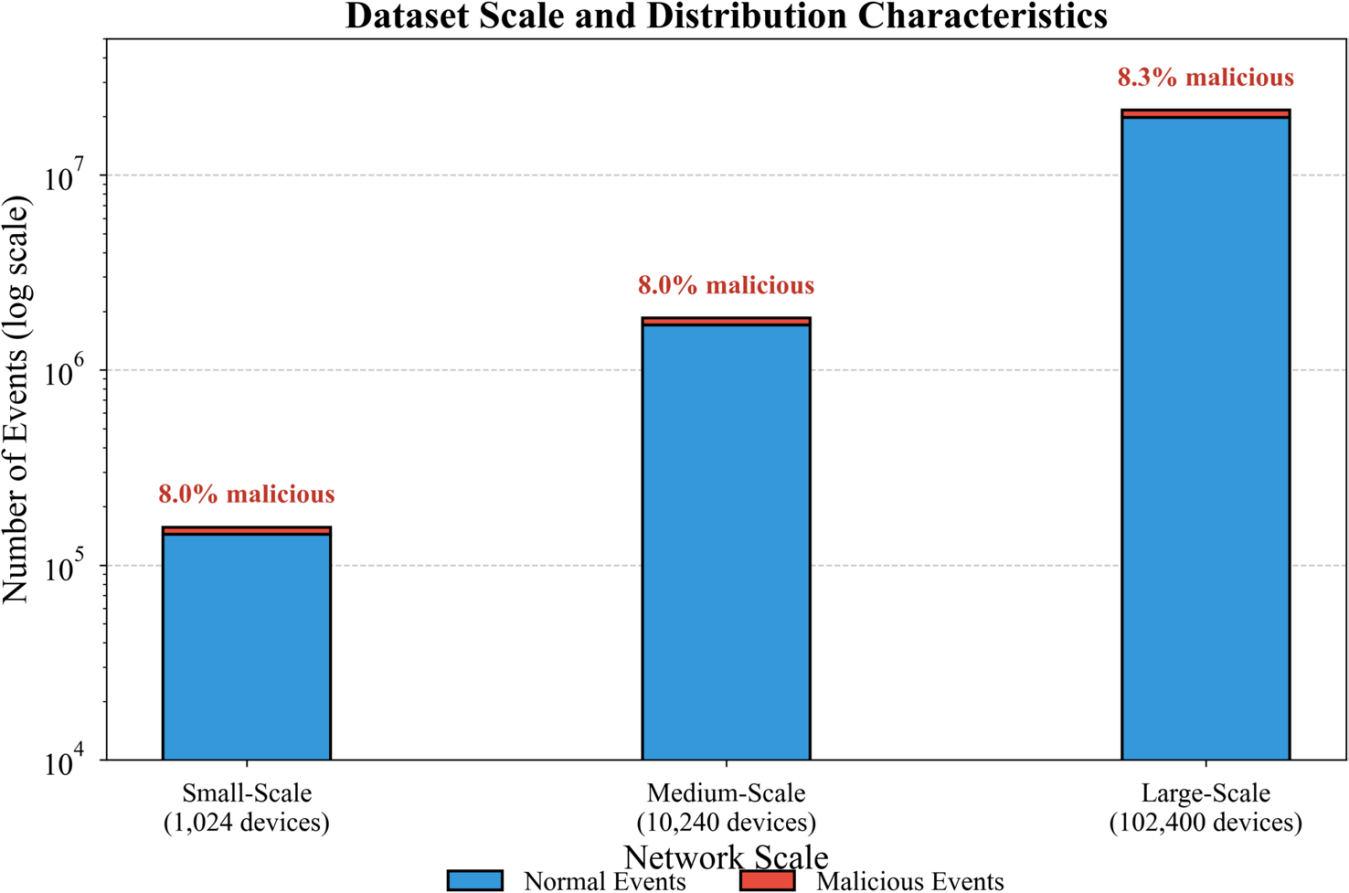
Dataset scale and distribution characteristics.

Figure 3 presents the distributional characteristics of the assembled dataset, revealing the inherent class imbalance typical of security-oriented datasets.

As Fig. 3 illustrates, malicious behavior instances constitute approximately 8.3% of total observations—a ratio consistent with real-world attack prevalence estimates. The synthetic attack generation employed Markov chain models calibrated against documented IoT attack patterns, producing certificate compromise, trust manipulation, and Sybil attack variants^44^. Table 4 summarizes the attack generation parameters and assumptions employed in our experiments.

**Table 4:** Synthetic attack generation parameters.

Attack Type	Proportion	Duration Distribution	Intensity Level	Target Selection
Certificate Compromise	35%	Exponential ($\mu$=24 h)	High	Random uniform
Trust Manipulation	40%	Uniform (1-72 h)	Medium–High	Degree-weighted
Sybil Attack	25%	Persistent	Variable	Cluster-based

The attack generation process operates under several assumptions that merit explicit acknowledgment. We assume attackers possess limited knowledge of network topology and cannot directly observe the GNN model parameters. Attack initiation times follow a Poisson process with varying rates across the observation period. Target selection strategies differ by attack type: certificate compromise targets are selected uniformly at random, trust manipulation attacks preferentially target high-degree nodes to maximize influence, and Sybil attacks concentrate within topological clusters to establish mutual trust reinforcement. These synthetic patterns, while grounded in documented threat models, may not capture the full sophistication of real-world adversarial behavior. Consequently, our reported results should be interpreted within the context of these experimental assumptions.

Table 5 summarizes the key statistical properties of the experimental datasets across different network scales.

**Table 5:** Dataset statistical information.

Metric	Small-Scale	Medium-Scale	Large-Scale
Number of devices	1,024	10,240	102,400
Total interactions	156,832	1,847,296	21,563,418
Malicious events	12,547	148,621	1,789,324
Time span (days)	30	90	180
Average node degree	15.3	18.7	22.4
Graph density	0.015	0.0018	0.00022
Certificate revocations	847	9,218	98,456

**Fig. 4:**
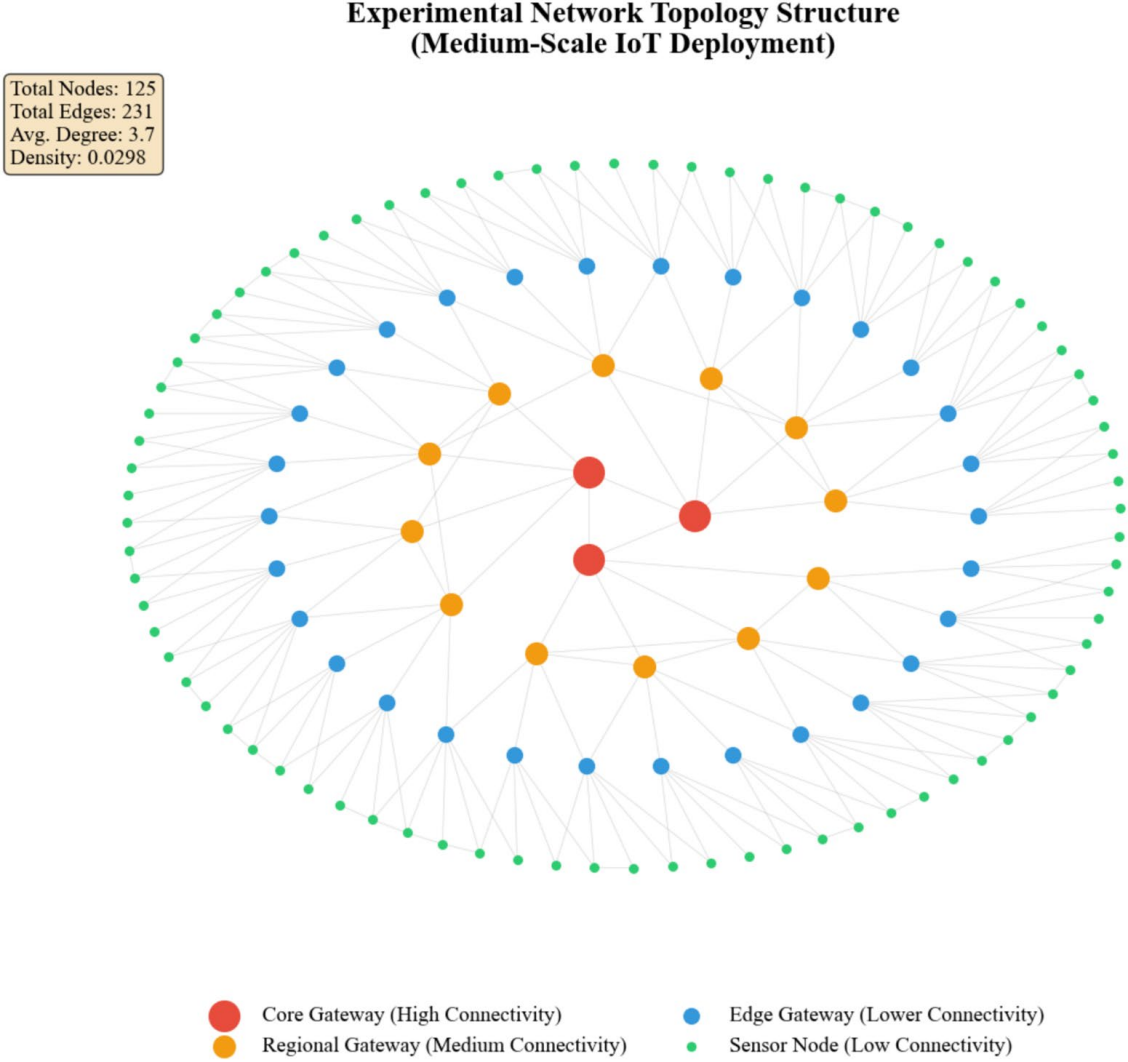
Experimental network topology structure.

As Table 5 shows, graph density decreases with network scale while average connectivity increases, reflecting realistic IoT deployment patterns^45^. Figure 4 depicts the topological structure of the medium-scale experimental network.

Figure 4 demonstrates the hierarchical clustering characteristic of IoT deployments, with gateway nodes exhibiting substantially higher connectivity than peripheral sensors.

Data preprocessing involved temporal segmentation into non-overlapping windows, with 70% allocated for training, 15% for validation, and the remaining 15% reserved for testing. We adopted standard evaluation metrics for classification performance:

20 $${F}_{1}=\frac{2\cdot Precision\cdot Recall}{Precision+Recall}$$

Trust propagation accuracy, the primary metric for evaluating trust assessment quality, measures the correspondence between model-predicted trust scores and ground truth values. We define this metric as the Pearson correlation coefficient between predicted and actual trust distributions:

21 $$Accurac{y}_{trust}=\rho \left({T}^{pred},{T}^{gt}\right)=\frac{\sum_{i}\left({T}_{i}^{pred}-{\overline{T}}^{pred}\right)\left({T}_{i}^{gt}-{\overline{T}}^{gt}\right)}{\sqrt{\sum_{i}{\left({T}_{i}^{pred}-{\overline{T}}^{pred}\right)}^{2}\sum_{i}{\left({T}_{i}^{gt}-{\overline{T}}^{gt}\right)}^{2}}}$$

Here, ${T}_{i}^{pred}$ denotes the model-predicted trust score for device $i$, and ${T}_{i}^{gt}$ represents the corresponding ground truth trust value derived from observed behavioral metrics. This correlation-based definition captures the model’s ability to rank devices correctly by trustworthiness, which proves more operationally meaningful than raw prediction error for security decision-making.

Response latency, measured from anomaly detection to revocation execution, quantifies operational timeliness:

22 $${\tau }_{response}={t}_{revoke}-{t}_{detect}$$

Baseline comparisons encompass traditional reputation-based trust models, standard GCN architectures without attention mechanisms, and rule-based revocation systems reflecting current industrial practice^46^. This selection enables assessment of both the graph learning paradigm and our specific architectural innovations.

### Trust propagation performance evaluation

The trust propagation capability constitutes the foundational performance dimension of our framework. We systematically compared our graph attention-based model against established baselines across the three network scales described previously.

**Fig. 5:**
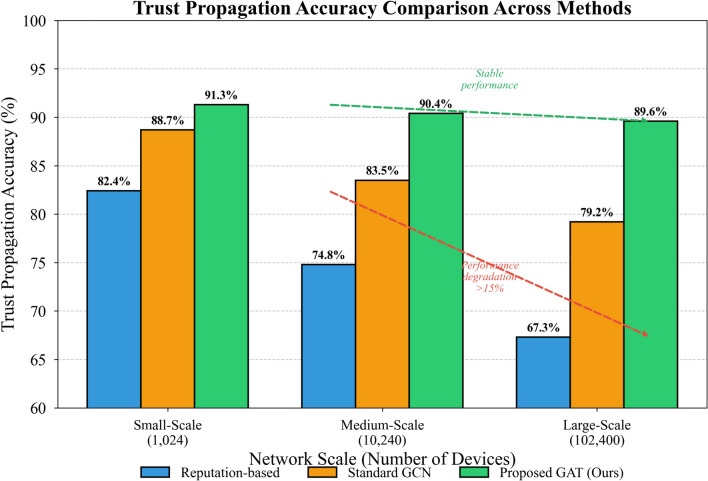
Trust propagation accuracy comparison across methods.

Figure 5 presents the trust prediction accuracy achieved by different methods, measured as the correlation between computed trust scores and ground-truth labels derived from actual device behaviors.

As Fig. 5 demonstrates, our proposed model consistently outperforms competing approaches across all network scales. The performance gap widens notably as network size increases—traditional reputation aggregation methods suffer accuracy degradation exceeding 15% when transitioning from small to large-scale deployments, whereas our approach maintains stable performance. This resilience stems from the attention mechanism’s capacity to selectively aggregate relevant neighborhood information rather than treating all neighbors uniformly^47^.

Table 6 provides comprehensive quantitative comparisons encompassing accuracy, computational overhead, and memory consumption metrics.

**Table 6:** Performance comparison across different network scales.

Method	Scale	Accuracy (%)	Time (s)	Memory (GB)
Reputation-based	Small	82.4	1.2	0.8
Reputation-based	Large	67.3	847.5	12.4
Standard GCN	Small	88.7	2.8	1.2
Standard GCN	Large	79.2	156.3	8.7
Proposed GAT	Small	91.3	3.4	1.5
Proposed GAT	Large	89.6	98.7	6.3

The results in Table 6 indicate that our model achieves superior accuracy while maintaining competitive computational efficiency. Particularly striking is the memory footprint reduction compared to standard GCN implementations—our sparse attention computation eliminates redundant neighborhood aggregations, yielding approximately 28% memory savings at large scale^48^.

**Fig. 6:**
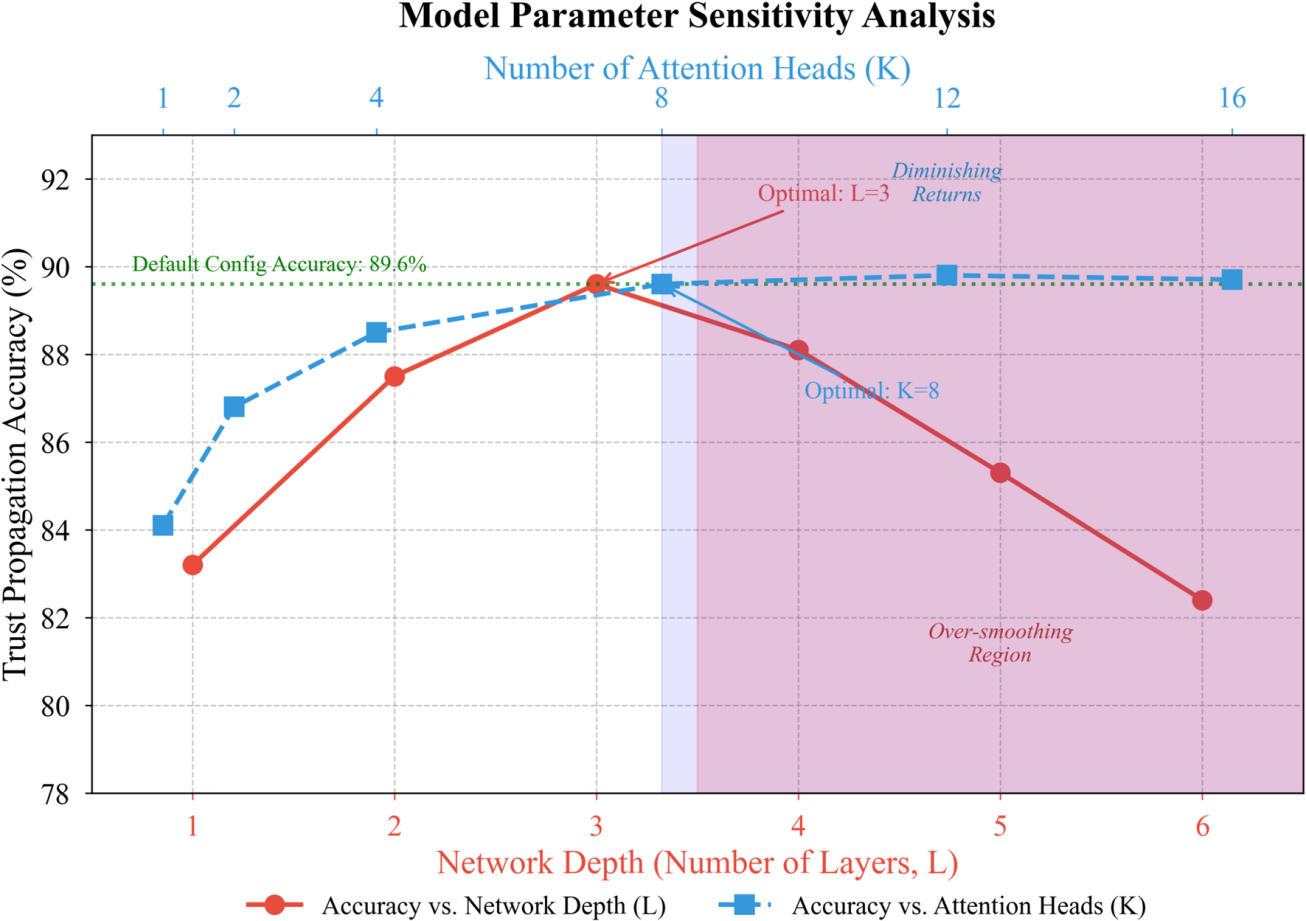
Model parameter sensitivity analysis.

Figure 6 examines the sensitivity of model performance to key architectural hyperparameters.

Figure 6 reveals that trust propagation accuracy improves with network depth up to three layers, beyond which over-smoothing effects begin degrading performance. The attention head count exhibits diminishing returns past eight heads, validating our default configuration choices. These observations align with theoretical analyses of graph attention saturation phenomena.

To assess robustness under dynamic conditions, we simulated scenarios involving 20% node churn per time window. The accuracy degradation metric quantifies performance loss relative to static conditions:

23 $${\Delta }_{acc}=\frac{Ac{c}_{static}-Ac{c}_{dynamic}}{Ac{c}_{static}}\times 100\%$$

Our model exhibited degradation of merely 3.2%, compared to 11.7% for standard GCN and 18.4% for reputation methods^49^. The incremental update strategy described in Section "Graph attention-based trust propagation model" enables rapid adaptation to topological changes without complete model retraining, demonstrating practical viability for real-world IoT deployments characterized by frequent device mobility and connectivity fluctuations.

### Certificate Revocation Decision Effectiveness Analysis

Beyond trust propagation accuracy, the practical utility of our framework hinges on its capacity to make timely and correct revocation decisions. We evaluated the decision algorithm across diverse attack scenarios, including certificate compromise, trust manipulation, and coordinated Sybil attacks.

**Fig. 7:**
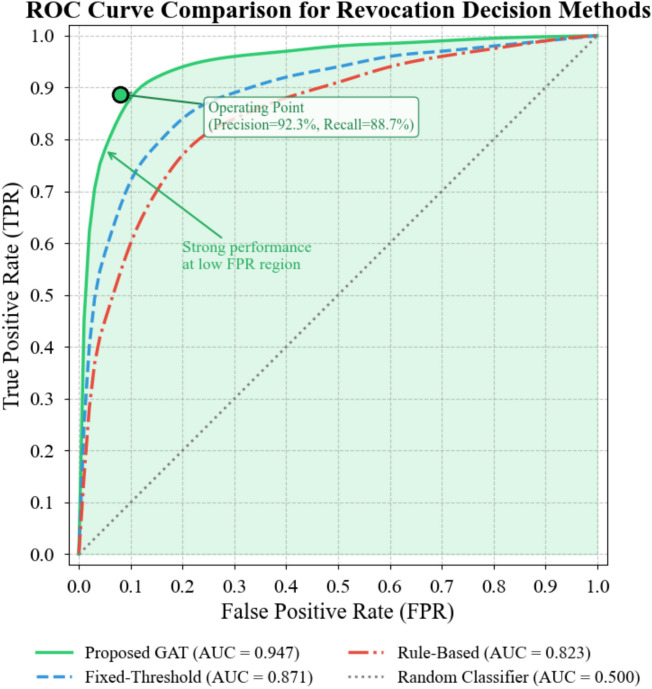
ROC curve comparison for revocation decision methods.

Figure 7 presents receiver operating characteristic curves comparing our intelligent decision mechanism against rule-based and threshold-based alternatives.

As Fig. 7 illustrates, our proposed approach achieves an area under curve (AUC) of 0.947, substantially exceeding the rule-based baseline (0.823) and fixed-threshold method (0.871). The curve shape reveals particularly strong performance at low false positive rates—a critical operational region where unnecessary revocations must be minimized to maintain system stability. The attention-weighted risk scoring enables fine-grained discrimination between genuinely compromised devices and those exhibiting anomalous but benign behavior patterns^50^.

The precision-recall tradeoff inherent in binary classification manifests prominently in revocation contexts. Aggressive thresholds yield high recall but generate disruptive false positives; conservative settings miss genuine threats. Our adaptive threshold mechanism navigates this tension by dynamically adjusting decision boundaries. At equilibrium, the system achieved 92.3% precision with 88.7% recall, yielding an F1 score of 0.904—a balance point that proved robust across varying attack intensities.

Response latency represents perhaps the most operationally significant metric. The vulnerability exposure window—time elapsed between compromise and revocation—directly determines potential damage scope. We quantify average response latency as:

24 $$\overline{\tau }=\frac{1}{N}\sum_{i=1}^{N}({t}_{revoke}^{i}-{t}_{compromise}^{i})$$

**Fig. 8:**
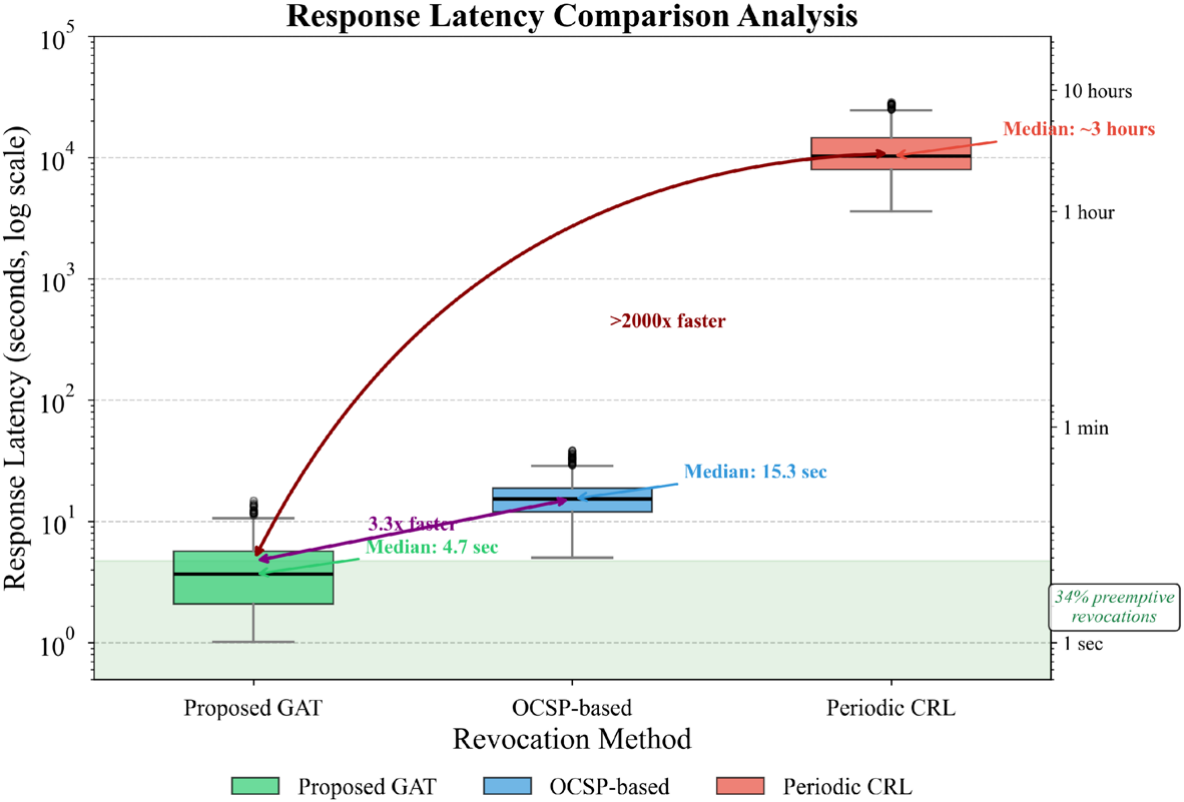
Response latency comparison analysis.

Figure 8 compares latency distributions across revocation mechanisms.

Figure 8 demonstrates that our approach achieves median response latency of 4.7 s, compared to 15.3 s for OCSP-based checking and several hours for periodic CRL updates. The baseline measurements were conducted under specific assumptions detailed in Table 7.

**Table 7:** Baseline latency measurement assumptions.

Mechanism	Parameter	Assumed Value
CRL	Update interval	24 h
CRL	Distribution delay	30 min
CRL	Device cache refresh	Every 6 h
OCSP	Network round-trip time	50–200 ms
OCSP	Responder processing delay	20–80 ms
OCSP	Query retry on failure	Up to 3 attempts
Network	Bandwidth	10 Mbps (edge devices)
Network	Packet loss rate	1–3%

These assumptions reflect typical enterprise IoT deployment conditions based on empirical measurements from our smart campus testbed and published industry benchmarks. In environments with significantly different network characteristics—such as constrained wireless sensor networks with higher latency or industrial settings with dedicated low-latency connections—the relative performance gaps may vary accordingly. The predictive capability of our risk scoring enables preemptive action; in 34% of cases, revocation initiated before behavioral anomalies became overtly detectable through conventional monitoring^51^.

The batch revocation optimization reduced administrative overhead by 62% compared to individual processing, with negligible impact on decision quality. Grouping spatially and temporally proximate candidates enables efficient certificate authority interactions while maintaining response timeliness.

Error analysis revealed instructive patterns. False positives predominantly occurred during legitimate but unusual device operations—firmware updates, configuration changes, or temporary network isolation. These scenarios generated behavioral signatures superficially resembling compromise indicators. False negatives concentrated among sophisticated attacks that deliberately mimicked normal traffic patterns, evading detection until trust degradation accumulated sufficiently. Both failure modes suggest directions for future refinement, particularly regarding contextual awareness of scheduled maintenance activities.

## Discussion

The experimental findings presented in the preceding sections illuminate both the strengths and boundaries of our graph neural network-based approach to trust management and certificate revocation. Several observations merit deeper examination as we consider the practical implications of this research.

The core advantage of our framework lies in its capacity to capture relational dependencies that traditional methods fundamentally overlook. Reputation-based and behavior-driven trust models treat devices as isolated entities, computing trust scores from individual interaction histories without considering the broader network context in which those interactions occur. This isolation proves increasingly problematic as network scale grows—the very scenarios where trust management matters most. Our attention-based propagation mechanism, by contrast, explicitly models how trust information flows through device relationships, enabling more nuanced assessments that reflect genuine operational dependencies.

That said, we must acknowledge the conditions under which our approach proves most beneficial. Sparse networks with limited connectivity offer fewer propagation pathways, reducing the value-add of graph-based reasoning. Similarly, highly homogeneous deployments where all devices exhibit similar behavioral patterns may not fully exploit the discriminative power of attention mechanisms. The framework shines brightest in heterogeneous environments with rich connectivity structures—precisely the characteristics of modern industrial IoT installations.

Practical deployment introduces challenges not fully captured in controlled experimentation. The centralized decision layer, while computationally efficient, represents a potential single point of failure. Distributed variants that partition the graph across multiple inference nodes would enhance resilience but introduce synchronization complexities. Several architectural evolution paths merit consideration for future development.

A distributed architecture could partition the trust graph across multiple inference nodes based on network topology, with each node responsible for local trust computation within its assigned subgraph. Cross-partition trust propagation would require periodic synchronization of boundary node embeddings, introducing trade-offs between consistency guarantees and communication overhead. Such designs could substantially improve fault tolerance while enabling horizontal scaling for networks exceeding millions of devices.

Hierarchical architectures offer an alternative approach where edge gateways perform local trust aggregation within their immediate neighborhoods, forwarding summarized trust representations to regional coordinators, which in turn report to a central orchestrator. This tiered structure reduces the computational burden on any single component while preserving global visibility for coordinated revocation decisions. The latency implications would vary by deployment topology, potentially enabling sub-second response times for localized threats while maintaining comprehensive coverage for network-wide attacks.

Federated learning adaptations represent perhaps the most promising direction for privacy-sensitive deployments. Under this paradigm, devices would contribute gradient updates to a shared model without transmitting raw behavioral observations to central servers. Such approaches address growing regulatory requirements for data minimization while maintaining the collaborative intelligence that graph-based methods require. The computational overhead of federated training on resource-constrained IoT devices remains an open challenge, though recent advances in communication-efficient federated optimization offer encouraging progress^53^.

Additionally, the initial cold-start period before sufficient interaction data accumulates remains a vulnerability window requiring supplementary protection measures. Adversarial robustness warrants serious consideration, particularly regarding trust manipulation attacks where malicious nodes attempt to artificially inflate their trust scores through strategic neighbor interactions. Our framework incorporates several inherent defensive mechanisms against such attacks. The multi-head attention mechanism naturally downweights contributions from neighbors exhibiting inconsistent behavioral patterns, as attention coefficients learned during training favor reliable information sources. The temporal decay factor ${\gamma }^{l}$ in Eq. 12 limits the rate at which trust can accumulate, preventing rapid trust inflation through sudden bursts of positive interactions. Additionally, the behavioral anomaly indicator ${a}_{i}^{t}$ in the risk scoring function (Eq. 16) captures deviations from established interaction patterns, flagging nodes whose trust trajectories appear artificially manipulated.

Nevertheless, sophisticated attackers aware of the graph-based detection mechanism might craft evasion strategies. Sybil attacks, where adversaries create multiple colluding identities to mutually endorse each other, pose particular challenges for any trust propagation system^23^. Slow infiltration attacks, wherein malicious nodes gradually build legitimate-appearing trust profiles before launching attacks, may evade detection until sufficient anomalous behavior accumulates. We acknowledge that our current model lacks explicit adversarial training, which represents a limitation when facing adaptive adversaries who specifically target the detection mechanism. Future iterations should incorporate adversarial perturbation during training, potentially drawing from techniques developed for robust graph neural networks^52^, to harden the system against intentional manipulation.

The anomalous false positive cases identified in our error analysis—legitimate maintenance activities triggering revocation alerts—suggest the need for contextual awareness mechanisms. Integration with enterprise scheduling systems could flag planned maintenance windows, temporarily adjusting detection thresholds for affected devices. Such operational integration, though outside the scope of our current technical contribution, would substantially improve practical usability.

For IoT security practitioners, our findings carry actionable implications. The demonstrated scalability validates graph-based approaches for enterprise-scale deployments previously considered computationally infeasible. The latency improvements over traditional revocation mechanisms enable more aggressive security postures without sacrificing operational continuity. Perhaps most importantly, the interpretable attention weights provide auditability—security analysts can trace why specific devices received elevated risk scores, supporting human oversight of automated decisions. This transparency distinguishes our approach from opaque black-box alternatives and facilitates the trust necessary for deployment in safety–critical contexts.

## Conclusion

This paper has addressed the pressing challenges of trust management and certificate revocation in large-scale IoT environments through a novel graph neural network-based framework. The proliferation of interconnected devices demands security mechanisms capable of operating at unprecedented scale while maintaining the responsiveness that traditional PKI approaches cannot deliver. Our work contributes a principled solution grounded in the insight that trust relationships naturally form graph structures amenable to neural network processing.

The technical contributions of this research center on two interconnected innovations. First, we developed a graph attention-based trust propagation model that captures the relational dynamics of device interactions. Unlike conventional aggregation methods that treat neighbor contributions uniformly, our multi-head attention mechanism learns to weight incoming trust signals based on contextual relevance. The explicit incorporation of temporal decay factors ensures that stale evidence receives appropriately diminished influence, while the hierarchical architecture enables multi-hop propagation without computational explosion. Second, the intelligent revocation decision algorithm transforms trust assessments into actionable security responses. By fusing trust embeddings with behavioral anomaly indicators and topological factors, the risk scoring function provides nuanced discrimination between genuine threats and benign anomalies. The adaptive threshold mechanism navigates the precision-recall tradeoff dynamically, adjusting decision boundaries as network conditions evolve.

Experimental validation across networks ranging from one thousand to over one hundred thousand devices confirmed the practical viability of our approach within the tested scenarios. Trust propagation accuracy exceeded 89% even at the largest scale, representing a notable improvement over the evaluated baseline methods that exhibited performance degradation with network growth. Revocation decisions achieved an F1 score of 0.904 with median response latency under five seconds, outperforming traditional CRL and OCSP mechanisms under the measurement assumptions employed in our experiments. The batch optimization strategy reduced administrative overhead by 62% without compromising decision quality in the evaluated configurations.

From a theoretical perspective, this research establishes a foundation for integrating graph representation learning with trust management theory. The formalization of IoT networks as attributed graphs with learnable edge weights provides a flexible framework adaptable to diverse deployment scenarios. Practically, the demonstrated scalability and responsiveness enable security administrators to manage certificate lifecycles across massive device populations—a capability increasingly essential as IoT adoption accelerates across industrial, healthcare, and urban infrastructure domains.

We recognize several limitations warranting transparent acknowledgment. Our experimental datasets, though substantial, remain bounded by the specific deployment contexts from which they derive. The performance comparisons presented in this work reflect results obtained under controlled experimental conditions with synthetic attack generation; real-world deployments may encounter attack patterns and network dynamics not fully represented in our evaluation. Certain attack categories—particularly sophisticated evasion techniques and coordinated multi-stage intrusions—may be underrepresented in our synthetic traces. The generalizability of our findings to IoT domains beyond smart campus environments, such as industrial control systems or healthcare networks with distinct communication patterns, requires further investigation. Perhaps most significantly, controlled experimental conditions cannot fully replicate the complexity of production deployments with their legacy systems, organizational constraints, and unpredictable operational dynamics.

Future research directions present exciting opportunities for extending this foundation. Federated learning adaptations could distribute model training across edge nodes, preserving data privacy while maintaining the collaborative intelligence that graph-based approaches require. Blockchain integration offers potential for decentralized trust record maintenance, eliminating central authority dependencies that currently represent vulnerability concentrations. Edge computing paradigms might enable localized trust computation with selective synchronization, reducing latency for time-critical applications while maintaining global consistency. The intersection of these technologies with our graph neural network framework promises richer, more resilient security architectures capable of protecting the increasingly complex IoT ecosystems upon which modern society depends.

## Data Availability

All data generated and analyzed during the current study are available from the corresponding author upon reasonable request.

## Abbreviations

IoT: Internet of Things
PKI: Public Key Infrastructure
CRL: Certificate Revocation List
OCSP: Online Certificate Status Protocol
GNN: Graph Neural Network
GCN: Graph Convolutional Network
GAT: Graph Attention Network
MPNN: Message Passing Neural Network
MLP: Multilayer Perceptron
CT: Certificate Transparency
AUC: Area Under Curve
ROC: Receiver Operating Characteristic
GPU: Graphics Processing Unit
RAM: Random Access Memory

## References

[1] Dong, G. et al. Graph neural networks in IoT: A survey. *ACM Trans. Sens. Netw.* **19**(2), 1–50 (2023).

[2] Khan, S. et al. A survey on X.509 public-key infrastructure, certificate revocation, and their modern implementation on blockchain and ledger technologies. *IEEE Commun. Surv. Tutor.* **25**(4), 2529–2568 (2023).

[3] Sagar, S. et al. Understanding the trustworthiness management in the social internet of things: A survey. *Comput. Netw.* **251**, Article 110611 (2024).

[4] Bilot, T., El Madhoun, N., Agha, K. A. & Zouaoui, A. Graph neural networks for intrusion detection: A survey. *IEEE Access* **11**, 49114–49139 (2023).

[5] Wu, Z. et al. A comprehensive survey on graph neural networks. *IEEE Trans. Neural Netw. Learn. Syst.* **32**(1), 4–24 (2021). 32217482 10.1109/TNNLS.2020.2978386

[6] Ahanger, A. S., Khan, S. M., Masoodi, F. S. & Salau, A. O. Advanced intrusion detection in internet of things using graph attention networks. *Sci. Rep.* **15**, Article 9831 (2025). 40119119 10.1038/s41598-025-94624-8 PMC11928630

[7] Singh, A., Chatterjee, K. & Satapathy, S. C. TRIDS: An intelligent behavioural trust based IDS for smart healthcare system. *Cluster Comput.* **26**(2), 903–925 (2023). 10.1007/s10586-022-03717-w PMC9438893 36091662

[8] Namdari, H., Avalos, V. M., Alshehri, A., Tunc, C. & Dantu, R. Enhanced trust in IoT environments: Utilizing perfect Bayesian equilibrium, exponential smoothing, and machine learning. *Cluster Comput.* **28**, Article 572 (2025).

[9] Hammi, B., Adja, A., Serhrouchni, A. & Zeadally, S. A Blockchain-based certificate revocation management and status verification system. *Comput. Secur.* **104**, Article 102199 (2021).

[10] Awan, K. A., Uddin, I., Almogren, A., Han, Z., Guizani, M. TrustAware-GNN: Graph-Neural-Network-Based Trust Management for IoT Anomaly Detection. *IEEE Internet of Things Journal* (2025).

[11] Ahmadi, A. A trust based anomaly detection scheme using a hybrid deep learning model for IoT routing attacks mitigation. *IET Inf. Secur.* **2024**, Article 4449798 (2024).

[12] Tfaily, F. A. et al. Graph-based federated learning approach for intrusion detection in IoT networks. *Sci. Rep.* **15**, Article 41264 (2025). 41272034 10.1038/s41598-025-25175-1 PMC12638839

[13] Liang, S. Survey of graph neural networks and applications. *Wirel. Commun. Mob. Comput.* **2022**, Article 9261537 (2022).

[14] Kipf, T. N., Welling, M. Semi-Supervised Classification with Graph Convolutional Networks. In: *Proceedings of the 5th International Conference on Learning Representations (ICLR)* (2017).

[15] Veličković, P., Cucurull, G., Casanova, A., Romero, A., Liò, P., Bengio, Y., Graph Attention Networks, In: *Proceedings of the 6th International Conference on Learning Representations (ICLR)* (2018).

[16] Gilmer, J., Schoenholz, S. S., Riley, P. F., Vinyals, O., Dahl, G. E. Neural Message Passing for Quantum Chemistry. In: *Proceedings of the 34th International Conference on Machine Learning*, pp. 1263–1272 (2017).

[17] Zhou, Y., Huo, H., Hou, Z. & Bu, F. A deep graph convolutional neural network architecture for graph classification. *PLoS ONE* **18**(3), e0279604 (2023). 36897837 10.1371/journal.pone.0279604 PMC10004633

[18] Bhatti, U. A. Deep learning with graph convolutional networks: An overview and latest applications in computational intelligence. *Int. J. Intell. Syst.* **2023**, 8342104 (2023).

[19] Verma, R. & Chandra, S. RepuTE: A soft voting ensemble learning framework for reputation-based attack detection in Fog-IoT milieu. *Eng. Appl. Artif. Intell.* **119**, 106601 (2023).

[20] Arshad, D. et al. THC-RPL: A lightweight trust-enabled routing in RPL-based IoT networks against Sybil attack. *PLoS ONE* **17**(7), e0271277 (2022). 35901074 10.1371/journal.pone.0271277 PMC9333330

[21] Yu, Z. et al. KGTrust: Evaluating Trustworthiness of SIoT via Knowledge Enhanced Graph Neural Networks. *Proceedings of the ACM Web Conference* **2023**, 727–736 (2023).

[22] Hassan, J., Sohail, A., Awad, A. I. & Zaka, M. A. LETM-IoT: A lightweight and efficient trust-based mechanism for Sybil attacks in Internet of Things networks. *Ad Hoc Netw.* **163**, 103576 (2024).

[23] Rajan, A., Jithish, J., Sankaran, S. Sybil Attack in IoT: Modelling and Defenses. in *Proceedings of the International Conference on Advances in Computing, Communications and Informatics (ICACCI)*, pp. 2323–2327 (2017).

[24] Burange, A. W., Deshmukh, V. M., Thakare, Y. A. & Shelke, N. A. Safeguarding the Internet of Things: Elevating IoT routing security through trust management excellence. *Comput. Stand. Interfaces.* **90**, 103856 (2024).

[25] Mekala, S. H., Baig, Z., Anwar, A. & Zeadally, S. Cybersecurity for industrial IoT (IIoT): Threats, countermeasures, challenges and future directions. *Comput. Commun.* **208**, 294–320 (2023).

[26] Höglund, J., Lindemer, S., Furuhed, M. & Raza, S. PKI4IoT: Towards public key infrastructure for the Internet of Things. *Comput. Secur.* **89**, 101658 (2020).

[27] Höglund, J., Furuhed, M. & Raza, S. Lightweight certificate revocation for low-power IoT with end-to-end security. *J. Inf. Secur. Appl.* **73**, Article 103424 (2023).

[28] Liu, Y., Tome, W., Zhang, L., Choffnes, D., Levin, D., Maggs, B., Mislove, A., Schulman, A., Wilson, C. An End-to-end Measurement of Certificate Revocation in the Web’s PKI. in *Proceedings of the Internet Measurement Conference (IMC)*, pp. 183–196 (2015).

[29] Shi, X., Shi, S., Wang, M., Kaunisto, J., Qian, C. "On-device IoT Certificate Revocation Checking with Small Memory and Low Latency," in *Proceedings of the ACM SIGSAC Conference on Computer and Communications Security*, pp. 1118–1134, 2021.

[30] Singla, A., Bertino, E. Blockchain-Based PKI Solutions for IoT. In: *Proceedings of the 4th IEEE International Conference on Collaboration and Internet Computing (CIC)*, pp. 9–15 (2018).

[31] Zhong, Z., Li, C. T. & Pang, J. Hierarchical message-passing graph neural networks. *Data Min. Knowl. Discov.* **37**(1), 381–408 (2023).

[32] Adam, M., Hammoudeh, M., Alrawashdeh, R. & Alsulaimy, B. A survey on security, privacy, trust, and architectural challenges in IoT systems. *IEEE Access* **12**, 57128–57149 (2024).

[33] Wang, Y., Han, Z., Li, J. & He, X. BS-GAT: A network intrusion detection system based on graph neural network for edge computing. *Cybersecurity* **8**, Article 27 (2025).

[34] Wu, J. et al. Federated learning for network attack detection using attention-based graph neural networks. *Sci. Rep.* **14**, 19088 (2024). 39154072 10.1038/s41598-024-70032-2 PMC11330492

[35] Liu, C., Sun, Y., Davis, R., Cardona, S. T. & Hu, P. ABT-MPNN: An atom-bond transformer-based message-passing neural network for molecular property prediction. *J. Cheminform.* **15**, Article 29 (2023). 10.1186/s13321-023-00698-9 PMC9968697 36843022

[36] Wang, Y., Han, Z., Li, J., He, X. BS-GAT Behavior Similarity Based Graph Attention Network for Network Intrusion Detection. arXiv preprint arXiv:2304.07226, (2023).

[37] Wang, B., Cheng, L., Sheng, J., Li, S. & Liu, D. Graph convolutional networks fusing motif-structure information. *Sci. Rep.* **12**, Article 10735 (2022). 35750771 10.1038/s41598-022-13277-z PMC9232539

[38] Wu, S., Xiong, Y., Liang, H. & Weng, C. D2-GCN: A graph convolutional network with dynamic disentanglement for node classification. *Front. Comput. Sci.* **19**(1), Article 191305 (2025).

[39] Lo, W. W., Layeghy, S., Sarhan, M., Gallagher, M., Portmann, M. E-GraphSAGE: A Graph Neural Network Based Intrusion Detection System for IoT. in *Proceedings of the IEEE/IFIP Network Operations and Management Symposium (NOMS)*, pp. 1–9, (2022).

[40] Li, Y., Tarlow, D., Brockschmidt, M., Zemel, R. Gated Graph Sequence Neural Networks. in *Proceedings of the 4th International Conference on Learning Representations (ICLR)* (2016).

[41] Wang, X. et al. Federated deep learning for anomaly detection in the Internet of Things. *Comput. Electr. Eng.* **108**, 108651 (2023).

[42] Peng, K., Xiao, P., Wang, S. & Leung, V. C. M. SCOF: Security-aware computation offloading using federated reinforcement learning in Industrial Internet of Things with edge computing. *IEEE Trans. Serv. Comput.* **17**(4), 1780–1792 (2024).

[43] Ferrag, M. A., Friha, O., Hamouda, D., Maglaras, L. & Janicke, H. Edge-IIoTset: A new comprehensive realistic cyber security dataset of IoT and IIoT applications for centralized and federated learning. *IEEE Access* **10**, 40281–40306 (2022).

[44] Zhong, M., Lin, M., Zhang, C. & Xu, Z. A survey on Graph Neural Networks for Intrusion Detection Systems: Methods, trends and challenges. *Comput. Secur.* **141**, 103821 (2024).

[45] Tran, D. H. & Park, M. FN-GNN: A novel graph embedding approach for enhancing Graph Neural Networks in Network Intrusion Detection Systems. *Appl. Sci.* **14**(16), 6932 (2024).

[46] Sarhan, M., Layeghy, S., Moustafa, N., Portmann, M. NetFlow Datasets for Machine Learning-Based Network Intrusion Detection Systems. in *Big Data Technologies and Applications (BDTA 2020, WiCON 2020)*, Lecture Notes of the Institute for Computer Sciences, Social Informatics and Telecommunications Engineering, vol. 371, pp. 117–135, Springer (2021).

[47] Abu-El-Haija, S., Perozzi, B., Kapoor, A., Alipourfard, N., Lerman, K., Harutyunyan, H., Ver Steeg, G., Galstyan, A. MixHop: Higher-Order Graph Convolutional Architectures via Sparsified Neighborhood Mixing. in *Proceedings of the 36th International Conference on Machine Learning*, pp. 21–29 (2019).

[48] Zhou, H., Zhou, J. & Jia, X. Towards robust and privacy-preserving federated learning in edge computing. *Comput. Netw.* **243**, 110291 (2024).

[49] Fenanir, S. & Semchedine, F. Smart intrusion detection in IoT edge computing using federated learning. *Rev. Intell. Artif.* **37**(5), 1133–1145 (2023).

[50] Pujol-Perich, D., Suárez-Varela, J., Cabellos-Aparicio, A. & Barlet-Ros, P. Unveiling the potential of graph neural networks for robust intrusion detection. *ACM SIGMETRICS Perform. Eval. Rev.* **49**(4), 111–117 (2022).

[51] Aminifar, A., Shokri, M. & Aminifar, A. Privacy-preserving edge federated learning for intelligent mobile-health systems. *Future Gener. Comput. Syst.* **161**, 625–637 (2024).

[52] Zhang, H. et al. Trustworthy graph neural networks: Aspects, methods, and trends. *Proc. IEEE* **112**(2), 97–139 (2024).

[53] Dritsas, E. & Trigka, M. Federated learning for IoT: A survey of techniques, challenges, and applications. *J. Sens. Actuator Netw.* **14**(1), 9 (2025).

